# Metabolomics and glucose tolerance in pregnancy and postpartum: The PONCH study

**DOI:** 10.1371/journal.pone.0335708

**Published:** 2025-11-07

**Authors:** Ulrika Andersson-Hall, Anders Bay Nord, Daniel Malmodin, Agneta Holmäng

**Affiliations:** 1 Institute of Neuroscience and Physiology, Department of Physiology, Sahlgrenska Academy, University of Gothenburg, Gothenburg, Sweden; 2 Swedish NMR Centre, University of Gothenburg, Gothenburg, Sweden; 3 National Bioinformatics Infrastructure Sweden (NBIS), Science for Life Laboratory, Sweden; Children's hospital of philadelphia, UNITED STATES OF AMERICA

## Abstract

**Aims:**

Pregnancy induces significant physiological changes, particularly important in obesity (OB) and gestational diabetes (GDM). We aimed to determine metabolite changes and their relation to clinical variables of obesity and glucose metabolism.

**Methods:**

Serum NMR metabolomics, clinical data, and body composition were determined in normoglycemic normal-weight (NW) (n = 32) and OB (n = 33) women at six time points spanning pregnancy and postpartum. Additionally, 31 GDM women (15 GDM-NW and 16 GDM-OB) were assessed during trimester 3.

**Results:**

Profound shifts in the metabolome during pregnancy were exemplified by decreased branched chain amino acids (BCAAs) and tyrosine, and increased phenylalanine, succinate, lactate, and pyruvate. Comparison with clinical variables showed strong correlation between BCAAs’ and bodyfat and insulin resistance mainly in the non-pregnant state. Conversely, pyruvate and lactate exhibited robust correlations with bodyfat, insulin resistance, and adipokines during pregnancy. Comparisons in late pregnancy showed higher levels of BCAAs, phenylalanine, lactate, and pyruvate in both obesity and GDM (GDM-NW and GDM-OB).

**Conclusions:**

BCAAs are elevated in obesity and GDM although may not be directly related to pregnancy-induced insulin resistance. Conversely, pyruvate and lactate appear connected to gestational changes of glucose metabolism where underlying obesity may contribute.

## Introduction

Pregnancy triggers large physiological changes, including changes such as the buildup of maternal fat reserves to fuel the latter stages of gestation and lactation [1]. Additionally, maternal insulin resistance increases to redirect glucose toward the rapidly developing fetus [2]. These pronounced physiological shifts are accompanied by alterations in various metabolites. Some metabolites have been studied extensively, while others remain less understood [3–5]. The intricate interplay between these changes in metabolites and maternal adaptations is an active area of research with potential to deepen our understanding of pregnancy-related metabolic dynamics.

Maternal obesity increases specific risks for the woman and the fetus, including conditions such as gestational diabetes (GDM), preeclampsia, delivery complications, and an extended susceptibility to obesity and type-2 diabetes for both the mother and the child [2]. Our prior research has explored the physiological responses to pregnancy in women with obesity compared to women of normal weight. Women with obesity demonstrated a lower increase in fat mass during pregnancy compared to women with normal weight. However, both groups of women displayed a similarly large increase in insulin resistance and alterations in levels of adipokines, such as increased leptin and soluble leptin receptor (LepR) [6]. Investigating metabolic shifts during and after pregnancy in women with obesity is important due to the increased risks to the mother and the child and the intergenerational transmitting cycle of obesity and metabolic disorders [7].

The increase in insulin resistance during pregnancy occasionally leads to GDM. Although a high BMI is a known predisposing factor, women with normal weight may also develop GDM [8]. The development of GDM can be attributed to a variety of factors, including pre-pregnancy high insulin resistance, inadequate compensatory insulin production by beta cells, or a combination of both these mechanisms.

In a previous metabolomics investigation [9], we examined alterations in women with GDM during the transition from pregnancy to the postpartum period. A gap remains in understanding metabolic changes during pregnancy for women or normal-weight or with obesity, and how these groups compare with metabolic profiles among women with GDM sharing similar BMI. While several studies have examined metabolic changes during pregnancy [3–5,10–15], few have taken a longitudinal approach encompassing both pregnancy and the postpartum period or compared obese and normal-weight populations. Furthermore, no metabolomics studies have investigated a GDM cohort subdivided into women with normal weight and those with obesity to further the understand the interplay of obesity and GDM. Our current objective is twofold: Firstly, to elucidate shifts in metabolite profiles in conjunction with clinical measurements throughout pregnancy and postpartum phases, comparing normoglycemic normal-weight and obese women. Secondly, to compare metabolite levels observed in late pregnancy with those of GDM women who share the same BMI categories. Results gained from our study could identify metabolites characteristic of GDM and/or obesity, which are distinct from those associated with normal pregnancy-induced insulin resistance and the postpartum period. These findings have the potential to contribute to early prediction and prevention strategies for GDM and postpartum hyperglycaemia, ultimately benefiting both maternal and child health.

## Methods

### Ethics

The study was approved by the Regional Ethical Review Board in Gothenburg (Dnr 402−08) and complied with relevant guidelines and regulations. Prior to their participation, all women were provided with both oral and written information about the study and subsequently signed informed written consent forms.

### Subjects and study design

Pregnant women of normal weight (NW: BMI 18.5–24.9 kg/m^2^: n = 32) or with obesity (OB: BMI ≥ 30 kg/m^2^: n = 33), were recruited by midwifes at six antenatal health units within the Gothenburg area as part of the Pregnancy Obesity Nutrition and Child Health (PONCH) study, as previously described [6,16]. The women attended three study visits during pregnancy (weeks 8–12 [trimester one, Tri1], 24–26 [two, Tri2] and 35–37 [three, Tri3]). If the women were willing to continue the study, three additional visits were conducted after pregnancy (6 months ± 10 days, 12 months ± 10 days and 18 months ± 10 days) at the Sahlgrenska University Hospital, Gothenburg. Exclusion criteria for NW and OB were being of non-European descent, having any form of diabetes mellitus (type-1-diabetes, type-2-diabetes or GDM), other chronic diseases or pregnancy related complications, use of tobacco or neuroleptic drugs and vegetarianism or veganism. Additionally, women diagnosed with GDM at the Sahlgrenska University Hospital were recruited during trimester two or three after their GDM diagnosis (GDM was diagnosed at 27 ± 7 gestational weeks) as part of the PONCH study as previously described [16], and attended an identical visit to NW and OB groups in Tri3.

Detailed descriptions of the visits have been previously described [6,16]. Briefly, all visits were conducted at the Sahlgrenska University Hospital in the morning after an overnight fast and included venous blood sampling, body composition measurements and completion of life-style questionnaires. The PONCH study enrolled a total of 136 NW, 73 OB and 55 GDM women during pregnancy. For this metabolomics study, a similar number of NW and OB subjects, 32 and 33 respectively, were randomly selected from the population that had stored serum samples and body composition measurements from both pregnancy and postpartum visits. In total, 67 NW had complete sample sets from Tri1–18 months postpartum, 39 OB had samples from pregnancy and at least one postpartum visit, of which 21 had samples up to 18 months. No significant differences were found between selected and non-selected women regarding BMI, body composition, or homeostatic model assessment of insulin resistance (HOMA-IR) versus their respective cohorts. Participant numbers remained consistent from Tri1–18 months postpartum for NW (n = 32); for the OB group, the numbers were n = 33 during pregnancy and 6 months postpartum but decreased to 31 at 12 months and 21 at 18 months. The reduced numbers in the OB group resulted from dropouts due to various reasons, including lack of time (n = 5), unrelated illness (n = 1), relocation from the area (n = 1), or exclusion due to starting a new pregnancy (n = 5). For comparison during late gestation, Tri3-samples from 31 GDM women were included in the analysis, with 15 categorized as normal weight (GDM-NW) and 16 with obesity (GDM-OB) at the start of pregnancy. Five GDM women were treated with insulin and two with metformin (medication distribution between GDM-NW and GDM-OB was not significantly different). All groups were recruited in parallel between 09-12-2009 and 31-01-2019.

### Clinical measurements

Body composition was assessed using air displacement plethysmography (BOD POD, software version 5.4.0, Cosmed), as previously described [6], including adjustments made for increased hydration of fat-free mass during pregnancy. Serum samples for NMR-analysis were immediately frozen and stored at -80C until analysis, which was performed for all selected samples simultaneously. Clinical blood analyses for HbA1c, glucose, insulin and high-sensitive C-reactive protein (hs-CRP) were conducted at the time of the study visit at the Clinical Chemistry Laboratory, Sahlgrenska University Hospital – accredited in accordance with the International Standard ISO 15189:2007. Leptin (Human Leptin Quantikine, R&D Systems, Minneapolis, MN; interassay coefficient of variation, 8.0% at 9 μg/l), the soluble leptin receptor (LepR) (Human Leptin R Quantikine, R&D Systems) and adiponectin (Human Adiponectin ELISA kit, Millipore, Billerica, MA; interassay coefficient of variation, 7.0% at 10.5 mg/l) were analysed simultaneously for all samples using ELISA. HOMA-IR was calculated as (fasting glucose × fasting insulin)/22.5 [17].

### NMR metabolomics

Sample preparation was done with a Bruker SamplePro Tube L liquid handler, essentially mixing equal amounts of buffer (75 mM sodium phosphate pH 7.4, 0.08% TSP-d_4_, 0.1% sodium azide in 20% v/v D_2_O) and sample and then transferring to SampleJet NMR tube racks, all done at 2°C. NMR data were acquired on a 600 MHz Bruker Avance III HD spectrometer equipped with a 5 mm TXI probe an a cooled SampleJet sample changer according to Bruker *in vitro* diagnostics for research standard operating procedures (IVDr; *Dona* et al., [18]). The B.I.Quant-PS 2.0.0 automated method (Bruker BioSpin), where the quantification is based on the use of an external calibration reference sample of known concentration (i.e., the ERETIC 2 approach [19]), was used to extract quantified metabolite profiles from the NMR data. To ensure stable performance, temperature, water suppression and shimming quality as well as the quantification reference are checked daily. The output of the automated analyses are 39 metabolites/small molecules. Metabolite concentrations were not normalized.

### Statistical analysis

Based on prior reports of changes in leucine and valine during pregnancy in women of normal weight or with obesity [20,21], power calculations indicated a minimum sample size of 6–26 women depending on amino acid and study (G*Power software, alpha = 0.05, power = 0.8). Multivariate analysis was performed using Simca 17 (Sartorius Data Analytics AB). Principal component analysis (PCA) was used to get a general overview of the data, while orthogonal partial least squares discriminant analysis (OPLS-DA) was used to discriminate between different groups. In the PCA analysis, 14 metabolites with several measurements under the detection limit were excluded to avoid the need of imputing values, resulting in 25 metabolites remaining.

As most clinical variables were not normally distributed, non-parametric testing was used for correlations between metabolites and clinical variables (Spearman analysis). P-values were adjusted for multiple testing using the Benjamini–Hochberg procedure to control the false discovery rate (FDR). Adjustments were performed separately for each clinical variable across the 17 metabolites of interest (FDR threshold 5%). Kruskal-Wallis analysis (with Mann-Whitney U post-hoc analysis corrected for multiple testing) was performed for comparison of normoglycemic women with GDM women of corresponding BMI, and separate post-hoc analysis of observed power was performed.

Group differences across timepoints for NW and OB women were analyzed using linear mixed-effects models of the form *Value ~ Group × Timepoint + (1|PersonID)* on 36 metabolites (3 being excluded due to being under the detection limit in almost all samples), with Timepoint as a categorical factor and random intercepts per participant. The analysis was performed using R (version 4.5.0). Models were estimated by REML with Kenward–Roger degrees of freedom. Post-hoc contrasts (P2–P1 at each timepoint) were adjusted for multiple comparisons using multivariate-t; if this failed, Benjamini–Hochberg FDR was applied.

To assess robustness, we performed sensitivity analyses including (i) random-slope models allowing participant-specific linear trends, (ii) complete-case analyses restricted to women with data at all six timepoints, and (iii) inverse probability weighting (IPW) to account for differential attrition across timepoints.

## Results

### Maternal clinical characteristics

Longitudinal clinical characteristics for start of pregnancy, late pregnancy and the first time-point after pregnancy for NW and OB women are summarized in Table 1. At the beginning of pregnancy, all clinical measurements, except fasting glucose, showed significant differences between NW and OB women. Following a substantial increase in insulin resistance during pregnancy in both groups (with a greater increase in OB than NW from Tri1 to Tri3, p < 0.001), fasting glucose was higher in OB compared to NW. This difference, along with other metabolic measurements, remained significant between NW and OB after pregnancy.

**Table 1:** Clinical characteristics for NW and OB women during and after pregnancy, and for GDM women during pregnancy.

		NW (n = 32)		OB (n = 33)		GDM (n = 31)		
		Mean	Std	Mean	Std	Mean	Std	P
Age		31.8	3.4	31.3	3.8	32.2	4.7	0.601
Parity (%) 0/1/2		37/47/16		49/39/12		61/29/10		0.465
BMI (kg/m^2^)	Tri 1	22.4	1.7	33.2	7.1			<0.001
	Tri 3	26.2	1.9	37.3	4.2	31.9	7.3	<0.001^abc^
	6 months	22.0	1.8	33.8	4.7			<0.001
Bodyfat (%)	Tri 1	26.5	5.0	46.1	4.7			<0.001
	Tri 3	28.4	4.8	44.2	4.7	37.8	10.2	<0.001^abc^
	6 months	26.7	5.9	46.1	6.3			<0.001
Glucose (mmol/L)	Tri 1	4.8	0.3	4.9	0.4			0.173
	Tri 3	4.5	0.3	4.8	0.4	5.1	0.9	0.001^ab^
	6 months	4.9	0.3	5.2	0.6			0.042
Insulin (mU/L)	Tri 1	4.8	1.6	12.7	5.5			<0.001
	Tri 3	7.6	3.1	21.0	8.4	14.9	8.8	<0.001^a^
	6 months	4.2	1.5	11.7	6.8			<0.001
HOMA-IR	Tri 1	1.0	0.4	2.8	1.3			<0.001
	Tri 3	1.5	0.7	4.5	1.9	3.6	2.8	<0.001^abc^
	6 months	0.9	0.4	2.7	1.8			<0.001
HbA1c	Tri 1	29.8	2.6	28.7	2.6			0.097
	Tri 3	29.8	2.6	31.1	2.7	34.1	4.1	<0.001^bc^
	6 months	33.2	2.7	31.6	2.1			0.110
hs-CRP (mg/L)	Tri 1	2.4	3.4	7.2	4.6			<0.001
	Tri 3	3.9	8.1	6.9	6.6	3.0	2.2	<0.001^ac^
	6 months	1.5	2.3	4.2	4.7			0.001
Leptin (ug/L)	Tri 1	12.3	6.3	49.9	17.0			<0.001
	Tri 3	16.9	12.0	56.5	25.1	31.8	22.3	<0.001^abc^
	6 months	6.3	4.0	31.6	16.0			<0.001
Adiponectin (mg/L)	Tri 1	18.2	7.7	10.8	6.0			<0.001
	Tri 3	13.0	5.5	9.7	3.7	12.4	6.9	0.060
	6 months	16.4	7.1	12.6	5.7			0.061
Leptin receptor (ug/L)	Tri 1	54.1	12.0	22.7	5.3			<0.001
	Tri 3	74.8	24.9	31.2	7.6	38.7	13.3	<0.001^abc^
	6 months	51.4	10.3	23.2	6.0			<0.001

*Statistical significance was determined with Kruskal Wallis test for continuous variables and Chi*^*2*^ *test for parity. Tri, trimester; NW, women of normal weight; OB, women with obesity; GDM, women with gestational diabetes; HOMA-IR, homeostatic model assessment of insulin resistance; hs-CRP, high-sensitivity C-reactive protein. Post-hoc Mann-Whitney-U tests were denoted with* ^*a*^*NW vs OB,* ^*b*^*NW vs GDM and* ^*c*^*OB vs GDM for p < 0.05.*

GDM women were only analysed in trimester 3. This group displayed BMI, body fat, insulin resistance, and adipokine levels intermediate to those of NW and OB women. Although glucose levels were higher in GDM women than in NW women, they were not significantly higher than in OB women.

### Metabolite changes during and after pregnancy

**Fig 1:**
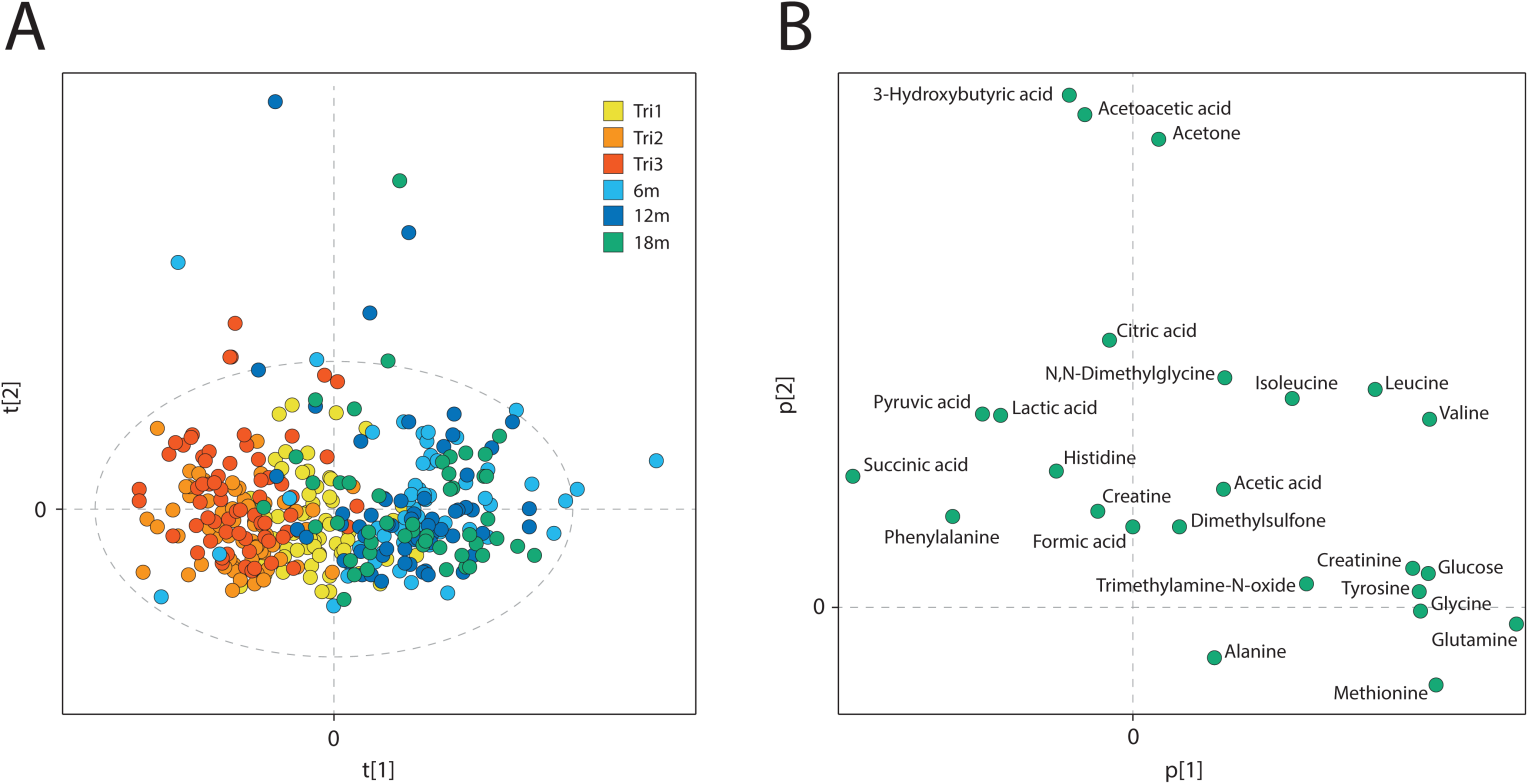
Metabolome at time-points during and after pregnancy in NW and OB women. The first two components in a PCA showing 362 measured NW and OB samples, after removal of one outlier, using 25 metabolite NMR concentration measurements. **A)** The first two scores (R^2^X(1)=18.4%, R^2^X(2)=12.3%, Q^2^ = 11.6%) shows a strong correlation with time rather than, e.g., individual or weight emphasizing the very large change during pregnancy, B) corresponding loading plot illustrates the major trends of the metabolites. Metabolites with increased concentrations during pregnancy are located on the left side of the loading plot with corresponding scores on the left side in the score plot, while metabolites with decreased concentrations are located to the right with corresponding scores on the right side of the plot. Some samples are very high in ketone bodies defining the direction of the second component.

Metabolite changes over the course of pregnancy and postpartum were analyzed for NW and OB women, who are all normoglycemic. A Principal Component Analysis (PCA) plot identified distinct directional changes across trimesters 1, 2, and 3, while postpartum samples (6–18 months) were closely clustered in the opposite direction, nearer to trimester 1 (Fig 1a). Metabolites primarily increased during pregnancy (left in Fig 1b) included succinate, pyruvate, lactate, and phenylalanine. Conversely, metabolites that decreased (right in Fig 1b) included glutamine, creatinine, lysine, branched-chain amino acids (valine, leucine, and isoleucine), glucose, glycine, tyrosine, methionine, and Trimethylamine-N-oxide.

### Distinct metabolic profiles in women with obesity compared to women of normal weight during and after pregnancy

#### Overall difference.

While the substantial changes during pregnancy may obscure the subtler variances attributed to BMI in a PCA, there were distinct metabolic profiles for NW versus OB women (S1 Fig). For example, a one component OPLS-DA model of all NW and OB samples having measured values for T1 to 12 months postpartum resulted in a good separation between the two groups despite the large changes during and after pregnancy (S1a Fig). The most prominent differences between NW and OB were higher branched chain amino acids (BCAAs; leucine, isoleucine and valine), aromatic amino acids (tyrosine and phenylalanine), alanine, histidine, lactate, pyruvate and creatine in the OB women (S1b Fig). The main common trend among the metabolites was that the difference between NW and OB stayed relatively the same during and after pregnancy. This was confirmed in individual OPLS-DA models at each time point (not shown), although some individual metabolites did not fit this pattern.

#### Associations with clinical measurements.

**Fig 2:**
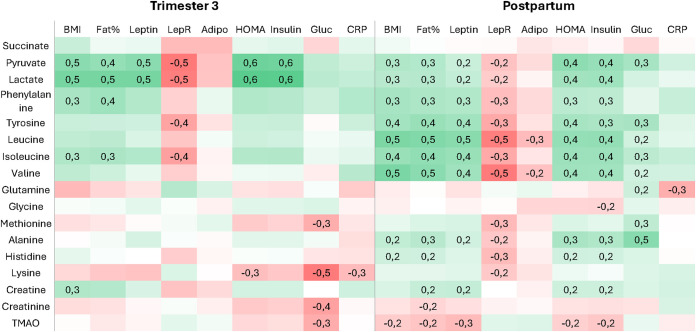
Heatmap of associations between clinical variables and metabolites of interest.

Given the differences in metabolic profiles between NW and OB women, a comparison with BMI and other clinical variables during late gestation and postpartum was conducted (Fig 2). Metabolites of interest were those identified in the analysis of changes during pregnancy or differences between NW and OB. The overall correlation patterns were similar during and after pregnancy, although fewer significant associations were observed during pregnancy, and some noteworthy differences emerged.

Pyruvate and lactate strongly correlated with BMI, body fat, leptin, LepR, HOMA, and insulin during pregnancy, and these correlations remained significant, albeit weaker, after pregnancy. In contrast, BCAAs displayed almost no correlations with clinical variables during pregnancy but were strongly associated with BMI, body fat, leptin, LepR, HOMA-IR, and glucose after pregnancy. Few metabolites, aside from pyruvate and lactate, were associated with HOMA-IR and insulin during late pregnancy, whereas the majority showed such correlations after pregnancy. For fasting glucose, the correlation pattern differed between time points, with no metabolites maintaining consistent associations from pregnancy to the postpartum state.

Metabolites were selected either because they showed relevant changes during pregnancy or because of notable differences between NW and OB women. The figure displays Spearman’s correlation coefficients (R) for associations between metabolites and clinical variables. Only correlations that remained significant after false discovery rate (FDR) correction (q < 0.05) are shown; non-significant cells are left blank. LepR, soluble leptin receptor; Adipo, adiponectin; HOMA, homeostatic model assessment of insulin resistance; Gluc, fasting glucose; CRP, high-sensitivity C-reactive protein; TMAO, trimethylamine N-oxide.

#### Longitudinal changes for metabolites in NW and OB women.

**Fig 3:**
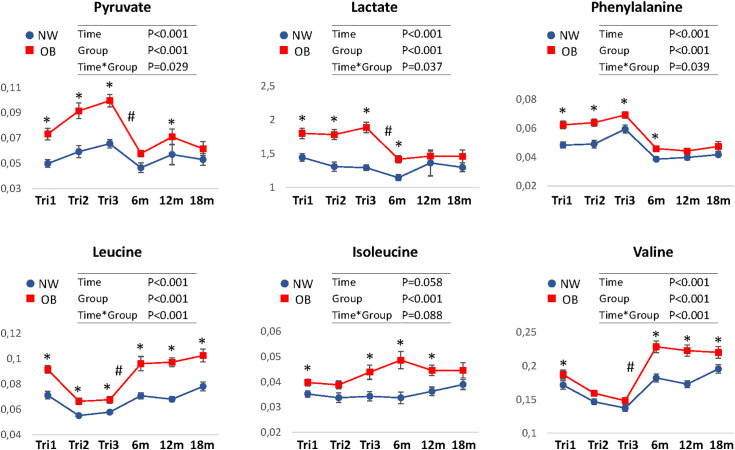
Longitudinal changes during and after pregnancy for selected metabolites. Concentrations in mM for NW (n = 32) and OB (n = 21-33) are shown as mean ± sem. Group differences across timepoints were analyzed using linear mixed-effects models with Group × Timepoint interaction. *p < 0.05 for NW vs. OB at the indicated timepoint (post-hoc contrasts, multiplicity-adjusted). For each metabolite, inset tables display global p-values from the mixed model for the main effects of Time, Group, and their interaction (Group × Timepoint).^#^ p < 0.05 NW vs OB för changes Tri3 to postpartum. NW, women of normal weight: OB, women with obesity; Tri1-3, trimester 1-3; 6-18m, 6-18 months postpartum.

Notable differences in pregnancy-induced metabolite changes between NW and OB women were observed (six selected metabolites shown in Fig 3, other mentioned metabolites in supplementary S2 Fig). Succinate, lactate, and pyruvate were higher during pregnancy compared with postpartum, with more substantial increases in OB compared with NW. BCAAs (leucine and valine), tyrosine, and methionine decreased during pregnancy, with the largest reductions occurring in OB women. Phenylalanine and creatine, on the other hand, were higher during pregnancy, with similar changes in both groups. Sensitivity analyses (random-slope, complete-case, and inverse probability weighting) yielded consistent results (S1 Table), supporting robustness against attrition and model misspecification.

### Comparison of NW and OB with GDM in late gestation

**Fig 4:**
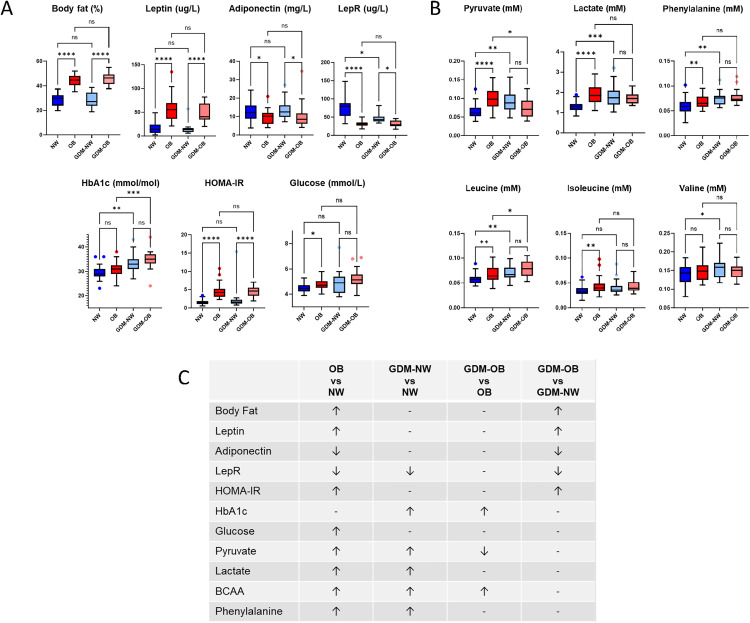
Comparison in trimester 3 between NW, OB, GDM-NW and GDM-OB women. Selected clinical measurements (A) and metabolites (B) are compared between NW (n = 33), OB (n = 32), GDM-NW (n = 15) and GDM-OB (n = 16) women. The boxes represent the 25^th^ to 75^th^ percentiles with Tukey whiskers and the line indicating median. *p < 0.05, **p < 0.01, ***p < 0.001 and ****p < 0.0001 for group comparisons using Kruskal-Wallis analysis and Mann-Whitney-U statistics adjusted for multiple comparisons. Observed power for Kruskal-Wallis analysis was ≥ 0.8 for all variables except one (Valine, power = 0.4) **C)** Overview of significant differences. NW, women of normal weight; OB, women with obesity; GDM-NW, women with GDM and of normal weight; GDM-OB, women with GDM and obesity; LepR, soluble leptin receptor; HOMA-IR, homeostatic model assessment of insulin resistance; BCAA, branched chained amino acids.

To explore the link between GDM and obesity, the trimester 3 clinical and metabolic variables of NW and OB cohorts were compared with a GDM cohort divided into GDM-NW and GDM-OB (Fig 4). As expected, body fat percentage aligned with BMI groups. Leptin, adiponectin, and insulin resistance differed based on BMI, but not on GDM status. Interestingly, soluble leptin receptor levels were notably elevated in NW women compared with all other groups, including GDM-NW. HbA1c levels followed GDM status rather than BMI. Fasting glucose exhibited a similar but non-significant trend, (possibly due to variability within the GDM groups). However, when comparing NW with the larger GDM group (Table 1) or with GDM-OB, the difference in glucose was significant (not shown). Pyruvate, lactate, phenylalanine, and leucine were significantly lower in NW compared to all other groups, including GDM-NW. Isoleucine and valine followed a similar trend but did not reach significance for all differences. Notably, none of these metabolites differed between GDM-NW and GDM-OB, indicating that the increase was related to both BMI and/or GDM status.

## Discussion

This longitudinal metabolomics study of pregnancy-related changes in both normal-weight and obese women confirmed previous findings, such as pregnancy-induced alterations in glucose and amino acid metabolism. It also provided clarity on previously unclear changes in metabolites like pyruvate, lactate, and succinate. The integration of clinical and metabolomics measurements during and after pregnancy has also unveiled partly new and intriguing results. Notably, branched-chain amino acids (BCAAs) exhibited a robust correlation with fat mass and insulin resistance in the non-pregnant state, as previously observed. Unexpectedly, this relationship weakened significantly during pregnancy. In contrast, lactate and pyruvate displayed a stronger association with fat mass and insulin resistance during pregnancy than in the non-pregnant state. Furthermore, the comparison of normoglycemic women with GDM women matched for BMI provided valuable insights into the complex relationship between clinical parameters and metabolite dynamics in late pregnancy. The findings shed light on how these parameters may interact in relation to GDM, obesity, or potentially both.

The observed changes in anthropometric and clinical variables align with earlier research conducted both within our group and by other researchers [6,22,23]. Increases in insulin resistance, leptin levels, and soluble leptin receptor levels, alongside declines in fasting glucose and adiponectin levels during pregnancy, were consistent with findings in both normal weight and obese cohorts in these studies. Our analysis extended to a comparison of trimester 3 data from women diagnosed with GDM who were categorized into normal-weight and obese groups. Notably, elevation in glucose and HbA1c levels were consistent across both obese and GDM groups. The significantly increased HOMA-IR and fat mass in the obese GDM women compared with the normal weight group underscores the well-established link between insulin resistance and body composition also in women with GDM.

As seen in previous studies, we observed a significant general shift in the metabolome during pregnancy [3,4,10,15]. An advantage of the current study is the inclusion of longitudinal timepoints both during and after pregnancy revealing predominantly unidirectional changes from trimesters 1–3, in contrast to postpartum measurements taken 6–18 months after childbirth, which all clustered tightly on the opposite side compared to trimester 1.

One of the more novel findings of our study was the consistent increase in pyruvate and lactate during pregnancy. Levels were higher in women with obesity and also elevated in GDM. In the normoglycemic women, pyruvate and lactate showed strong associations with BMI, body fat percentage, leptin, soluble leptin receptor, and HOMA-IR during pregnancy, whereas most other metabolites displayed such associations only in the non-pregnant state. This pattern suggests that lactate and pyruvate may be particularly linked to the metabolic adaptations of pregnancy.

A plausible explanation is that elevated lactate and pyruvate partly reflect the well-documented rise in hepatic gluconeogenesis in late gestation, which increases maternal glucose production to meet fetal and placental demands [24]. Our previous research has also shown that higher pyruvate levels after GDM are associated with an increased risk of prediabetes and type 2 diabetes [25], underlining their possible long-term relevance.

We also observed a novel increase in succinate during pregnancy. Unlike lactate and pyruvate, this rise did not differ between NW and OB women and showed no associations with anthropometry or insulin resistance. Nevertheless, findings from non-pregnant studies indicate that succinate may act as a signalling metabolite influencing insulin secretion [26] and obesity-induced inflammation [27], and expression of the succinate receptor SUCNR1 has been demonstrated in human placenta [28].

Though these mechanisms have not been directly investigated during pregnancy, our observations are consistent with emerging evidence from non-pregnant and in vitro studies showing that pyruvate, lactate, and succinate can act as regulators of glucose and energy metabolism, as well as markers of type 2 diabetes risk [26,27,29–33]. Their roles may be linked not only to their function as metabolic intermediates but also to receptor-mediated signalling pathways, including G-protein coupled receptors, that influence insulin sensitivity, insulin secretion, and inflammatory responses.

In addition to changes in glycolytic intermediates, we observed alterations in the creatine/phosphocreatine system. This pathway contributes to ATP buffering through creatine kinase activity, and elevated creatine levels in our cohort — particularly in women with obesity — may reflect the high energy demands of pregnancy, most pronounced in the insulin-resistant state. Similar findings of altered energy metabolism during pregnancy in insulin-resistant subjects have recently been reported in an NMR study by Liu et al [34].

In agreement with prior research, our study showed that during pregnancy, levels of BCAAs and tyrosine were lower, whereas phenylalanine levels were higher in comparison to the postpartum state [3,12,14]. Notably, the increase in BCAAs following pregnancy was most pronounced in women with obesity and we observed robust associations between BCAAs and clinical variables such as fat mass and insulin resistance after pregnancy. This finding aligns with previous investigations demonstrating associations between elevated BCAAs and insulin resistance in non-pregnant individuals [30,35]. Interestingly, the associations between BCAAs and fat mass or insulin resistance were weak or absent during pregnancy. This suggests that BCAAs may have a smaller role in the naturally occurring increase in insulin resistance during pregnancy. When examining the absolute values of BCAAs in the third trimester in women of normal weight with or without GDM, we identified elevated levels of leucine and valine in women with GDM. This suggests that high BCAAs could potentially serve as an indicator for risk of GDM development independent of pregnancy-induced changes in insulin resistance. It is worth noting that, similar to pyruvate, our previous research has highlighted the association between elevated BCAA levels post-GDM pregnancies and an increased risk of developing T2D[25].

Intriguing complexities arise from the patterns observed in adipokines during late pregnancy. Leptin and adiponectin levels mirrored variations in BMI, whereas the soluble leptin receptor consistently exhibited reduced levels in groups characterized by high BMI and/or GDM. This intriguing pattern supports our previous research, in which we proposed that the modulation of leptin bioavailability through the soluble receptor might play a critical role in optimizing fat mass and insulin sensitivity during pregnancy [6]. Moreover, this regulatory mechanism may extend its influence to infant fat mass accumulation, potentially contributing to early metabolic programming [36]. These findings underscore the noteworthy pregnancy-related associations of pyruvate and lactate with the soluble leptin receptor in our current study, underlining the importance of exploring this topic in future research.

The PONCH study employs a well-controlled longitudinal approach that involves six identical participant visits for NW and OB women, encompassing clinical blood and body composition measurements. Prior pregnancy studies have either showed increases or no changes in lactate, pyruvate and succinate [4,11–15]. Lactate seems to be increased very early in pregnancy and both pyruvate and succinate show increases between trimester 1 and 2, which means that studies measuring changes late in pregnancy or lacking non-pregnant matched samples may miss this development. Furthermore, our results indicate that both pyruvate and lactate increase most in women with obesity, underscoring the relevance of a study population which consider weight and body composition. Comparisons can also be made between the NW and OB groups and women with GDM who attended an identical PONCH visit in the third trimester, although the number of women in GDM groups are relatively small. It is crucial to note that all serum samples were collected from fasting individuals and handled identically by the same clinical study unit before freezing. Sampling in the fasted state is essential when drawing conclusions from metabolite levels, particularly in the context of energy and glucose metabolism, and the identical sample preparation process is critical in ensuring the production of high-quality metabolomics data.

We consider the prospective design and careful phenotyping to be strengths of the study. Nevertheless, there is the potential for bias related to diet and lifestyle due to the primary focus on these factors during recruitment. For instance, the NW group exhibited lower than average gestational weight gain compared to similar studies involving Scandinavian women. In contrast, OB women displayed gestational weight gain levels comparable to those observed in previous studies [37,38]. Furthermore, after childbirth, participants in both groups did not retain the additional body weight gained during pregnancy, in contrast to the typical trend observed in the broader Swedish population [6,39]. Another limitation is that a small subset of women with GDM received pharmacological treatment (five with insulin and two with metformin). These women still exhibited higher glucose levels than untreated GDM participants (data not shown), and insulin treatment did not normalize metabolite profiles. While the distribution of treated women did not differ between the GDM subgroups defined by BMI, the comparison of GDM groups with their BMI-matched controls may be influenced by the presence of pharmacological treatment. Given the small numbers, adjustment for treatment was not feasible, but this potential source of heterogeneity should be acknowledged.

### Conclusion

In conclusion, this longitudinal metabolomics study deepens our understanding of metabolic changes during pregnancy, highlighting both previously shown and novel changes. The increases in lactate and pyruvate during pregnancy, along with their robust association with pregnancy-induced clinical changes, as well as their increase in both obesity and GDM, suggest they play important roles in glucose metabolism during pregnancy. Furthermore, absolute levels of BCAAs were linked to obesity and GDM, although they did not exhibit strong associations with pregnancy-induced clinical changes in normoglycemic women. Further exploration of these findings is warranted to better understand the complex mechanisms underlying the physiological changes of pregnancy to improve the health of mothers and their offspring.

## Supporting information

S1 Fig Metabolic differences between women of normal-weight or with obesity. A one component OPLS-DA model of 32 NW individual’s 160 Tri1-PP12 samples vs 23 OB individual’s 115 samples, R2 = 0.42 and Q2 = 0.37, well above any values from 999 permutations with largest R2 = 0.12 and Q2 = 0.05, using seven cross validation groups with samples from the same person in the same group. A) Score plot showing that the single component model discriminates the groups, being above or below the x-axis, approximately equally well independent of time during the pregnancy The samples are ordered from left to right in five blocks Tri1-12m each block showing NW first then OB, with individuals ordered the same in each block. B) Loading plot showing higher BCAAs, aromatic amino acids, alanine, histidine, lactate, pyruvate and creatine in the OB women. The error bars show the Jack knife standard error of the loading computed from all rounds of cross validation.
(PDF)

S2 Fig Longitudinal changes during and after pregnancy for selected metabolites. Concentrations in mM for NW (n = 32) and OB (n = 21–33) are shown as mean ± sem. Group differences across timepoints were analyzed using linear mixed-effects models with Group × Timepoint interaction. *p < 0.05 for NW vs. OB at the indicated timepoint (post-hoc contrasts, multiplicity-adjusted). For each metabolite, inset tables display global p-values from the mixed model for the main effects of Time, Group, and their interaction (Group × Timepoint). ^#^ p < 0.05 NW vs OB för changes Tri3 to postpartum. NW, women of normal weight: OB, women with obesity; Tri1–3, trimester 1–3; 6-18m, 6–18 months postpartum.
(PDF)

S1 Table Sensitivity analyses for metabolites. (DOCX)
