# Supplementary material for: Metabolomics and glucose tolerance in pregnancy and postpartum: The PONCH study
Source: PLoS One. 2025 Nov 7;20(11):e0335708. doi: 10.1371/journal.pone.0335708 (PMC12594331; doi:10.1371/journal.pone.0335708)
Supplement: S1 Fig — A one component OPLS-DA model of 32 NW individual’s 160 Tri1-PP12 samples vs 23 OB individual’s 115 samples, R2 = 0.42 and Q2 = 0.37, well above any values from 999 permutations with largest R2 = 0.12 and Q2 = 0.05, using seven cross validation groups with samples from the same person in the same group. A) Score plot showing that the single component model discriminates the groups, being above or below the x-axis, approximately equally well independent of time during the pregnancy The samples are ordered from left to right in five blocks Tri1-12m each block showing NW first then OB, with individuals ordered the same in each block. B) Loading plot showing higher BCAAs, aromatic amino acids, alanine, histidine, lactate, pyruvate and creatine in the OB women. The error bars show the Jack knife standard error of the loading computed from all rounds of cross validation. (PDF) [file pone.0335708.s001.pdf]

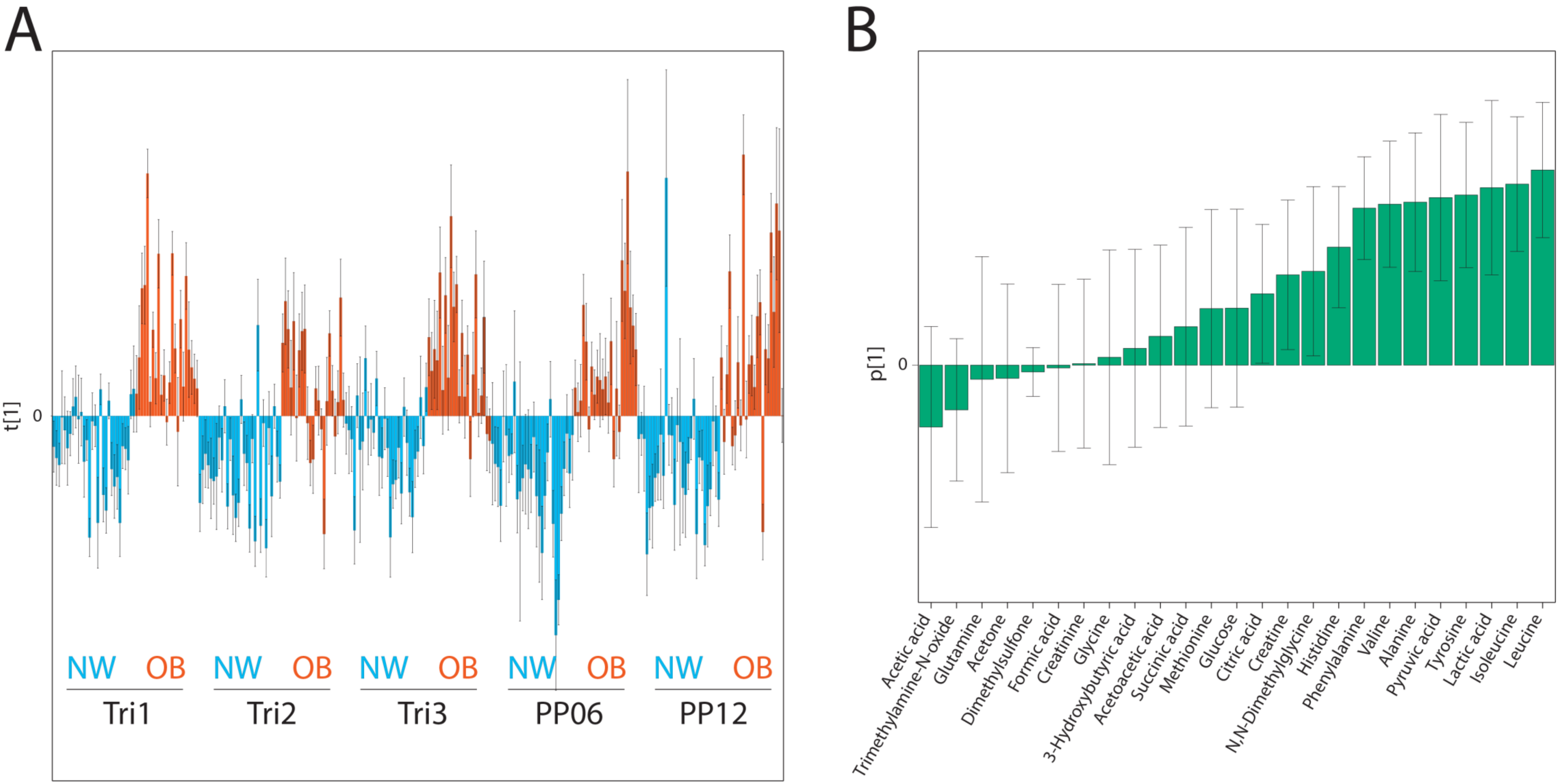

S1 Fig. Metabolic differences between women of normal-weight or with obesity. A one component OPLS-DA model of 32 NW individual's 160 Tri1-PP12 samples vs 23 OB individual's 115 samples, R2Y = 42% and Q2 = 37%, well above any values from 999 permutations with largest R2Y=12% and Q2 = 5% (p = 2*10-25 from CV-ANOVA analysis), using seven cross validation groups with samples from the same person in the same group. A) Score plot showing that the single component model discriminates the groups, being above or below the x-axis, approximately equally well independent of time during or after the pregnancy The samples are ordered from left to right in five blocks Tri1-12m each block showing NW first then OB, with individuals ordered the same in each block. B) Loading plot showing higher BCAAs, aromatic amino acids, alanine, histidine, lactate, pyruvate and creatine in the OB women. The error bars show the Jack knife standard error of the loading computed from all rounds of cross validation. Tri, trimester; PP06, postpartum 6 months; PP12, postpartum 12 months.
