# Supplementary material for: Metabolomics and glucose tolerance in pregnancy and postpartum: The PONCH study
Source: PLoS One. 2025 Nov 7;20(11):e0335708. doi: 10.1371/journal.pone.0335708 (PMC12594331; doi:10.1371/journal.pone.0335708)
Supplement: S2 Fig — Concentrations in mM for NW (n = 32) and OB (n = 21–33) are shown as mean ± sem. Group differences across timepoints were analyzed using linear mixed-effects models with Group × Timepoint interaction. *p < 0.05 for NW vs. OB at the indicated timepoint (post-hoc contrasts, multiplicity-adjusted). For each metabolite, inset tables display global p-values from the mixed model for the main effects of Time, Group, and their interaction (Group × Timepoint). # p < 0.05 NW vs OB för changes Tri3 to postpartum. NW, women of normal weight: OB, women with obesity; Tri1–3, trimester 1–3; 6-18m, 6–18 months postpartum. (PDF) [file pone.0335708.s002.pdf]

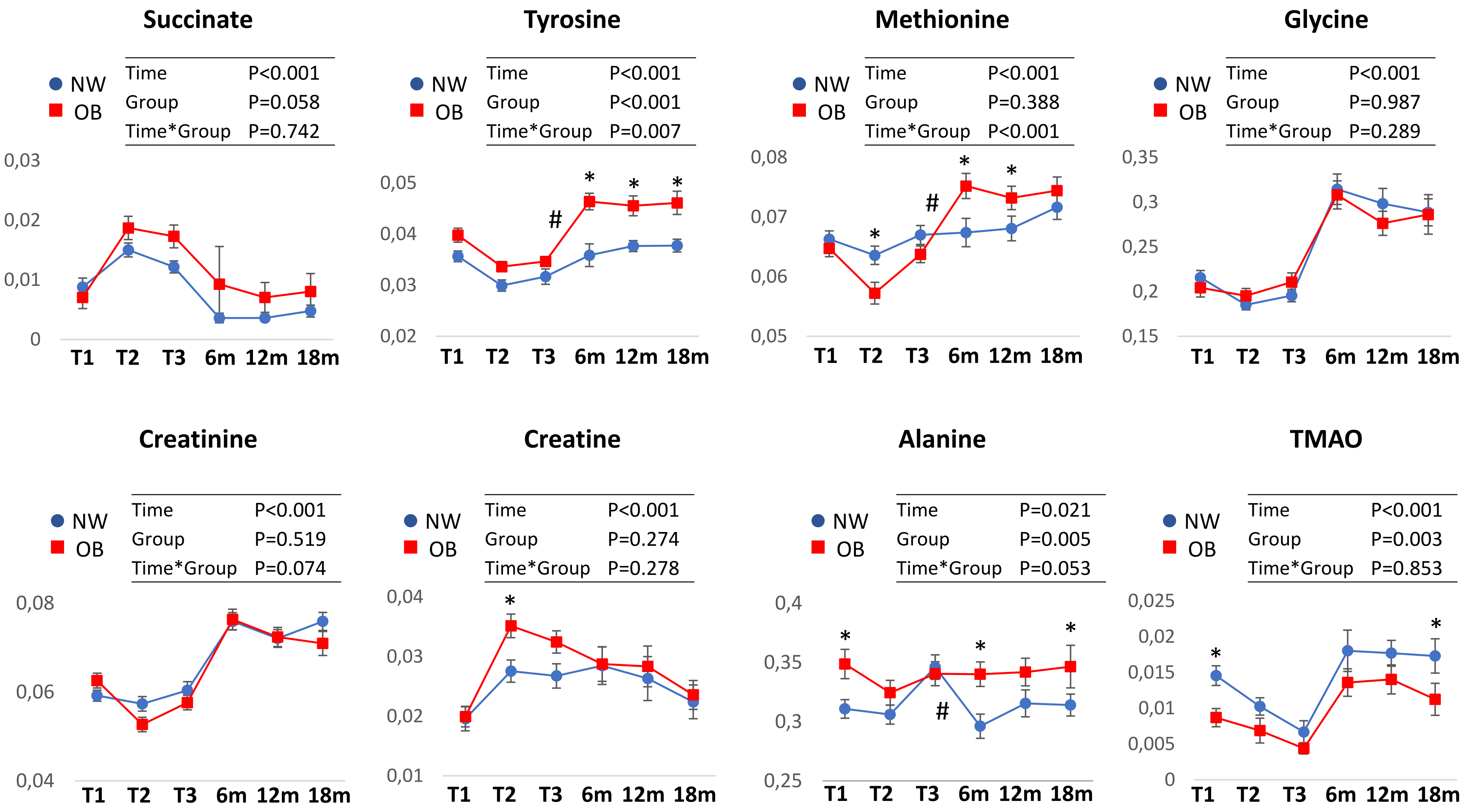

S2 Fig. Longitudinal changes during and after pregnancy for selected metabolites. Concentrations in mM for NW (n=32) and OB (n=21-33) are shown as mean ± sem. Group differences across timepoints were analyzed using linear mixed-effects models with Group × Timepoint interaction. *p < 0.05 for NW vs. OB at the indicated timepoint (post-hoc contrasts, multiplicity-adjusted). For each metabolite, inset tables display global p-values from the mixed model for the main effects of Time, Group, and their interaction (Group × Timepoint). [#] p<0.05 NW vs OB för changes Tri3 to postpartum. NW, women of normal weight: OB, women with obesity; Tri1-3, trimester 1-3; 6-18m, 6-18 months postpartum.
