# Supplementary material for: Metabolomics and glucose tolerance in pregnancy and postpartum: The PONCH study
Source: PLoS One. 2025 Nov 7;20(11):e0335708. doi: 10.1371/journal.pone.0335708 (PMC12594331; doi:10.1371/journal.pone.0335708)
Supplement: S1 Table — (DOCX) [file pone.0335708.s003.docx]

# S1 Table. Sensitivity analyses for metabolites.

| **Metabolite** | **Base model p** | **Complete-case p** | **IPW p** | **Stable** |
| --- | --- | --- | --- | --- |
| Methionine | 4.3e-05 | 0.006431 | 4.7e-05 | Yes |
| Valine | 0.000123 | 0.027738 | 0.000152 | Yes |
| Lysine | 0.000353 | 0.006794 | 0.000277 | Yes |
| Leucine | 0.001262 | 0.003945 | 0.00147 | Yes |
| Tyrosine | 0.007451 | 0.065617 | 0.006695 | Yes |
| Pyruvate | 0.029177 | 0.25414 | 0.022927 | Yes |
| Lactate | 0.036546 | 0.077189 | 0.02927 | Yes |
| Phenylalanine | 0.039497 | 0.585186 | 0.037365 | Yes |
| Alanine | 0.052685 | 0.114383 | 0.055272 | Yes |
| Glutamine | 0.058015 | 0.248928 | 0.05014 | Yes |
| Creatinine | 0.073571 | 0.012198 | 0.062963 | Yes |
| Isoleucine | 0.088105 | 0.466403 | 0.08549 | Yes |
| Creatine | 0.277754 | 0.345942 | 0.288851 | Yes |
| Glycine | 0.2885 | 0.623188 | 0.301505 | Yes |
| Succinate | 0.741832 | 0.005103 | 0.753315 | Yes |
| TrimethylamineNoxide | 0.853074 | 0.126433 | 0.836874 | Yes |
| Histidine | 0.952152 | 0.969145 | 0.948964 | Yes |

*Shown are p-values from the base linear mixed-effects model, complete-case (CC) analyses and inverse probability weighting (IPW) and a stability indicator (Stable = consistent significance pattern between base and IPW). Maximum weight applied in IPW was 2.06 for all metabolites. Stability of results across approaches supports robustness against attrition and model misspecification.*
